# Fiberoptic endoscopic evaluation of swallowing: where should we go from here?

**DOI:** 10.1590/2317-1782/e20250350en

**Published:** 2026-06-01

**Authors:** Thaís Coelho Alves, Suely Mayumi Motonaga Onofri, Roberta Gonçalves da Silva

**Affiliations:** 1 Laboratório de Disfagia, Departamento de Fonoaudiologia, Universidade Estadual Paulista – UNESP - Marília (SP), Brasil.

Fiberoptic endoscopic evaluation of swallowing (FEES), proposed by Langmore et al. in 1988^(1)^, revolutionized instrumental swallowing assessment by providing practical advantages and significantly contributing to clinical decision-making in oropharyngeal dysphagia. Since then, numerous studies have described — albeit in a non-standardized protocol — the methods of execution, the use of dyes, volumes, and utensils, as well as the scales for analysis and measurement^(2-4)^. If there is a consensus in the literature, it is the absence of a universal protocol^(5)^. Study populations, contexts of application, and, above all, the experience of the professional who performs and interprets the exam are all heterogeneous. Thus, they directly impact the possibilities of standardization and the genuine contributions of FEES to the diagnosis and rehabilitation of oropharyngeal dysphagia.

Having been consolidated for decades, we know how to perform the test^(6-8)^. However, a recurring theme in scientific conferences and publications is the continued exploration of the same fundamental points, despite the field's technical and scientific maturity. Criticism is needed: the insistence on debating how to do it has overshadowed the question that truly matters at this moment: “What to do with what we find?”. Therefore, this authors’ comment aims to encourage readers to reflect on the necessary change in FEES perspectives in a direction that truly impacts clinical decision-making across different patient profiles.

We currently observe an academic world that continuously revolves around the same thematic axes without any substantial progress, including whether dye is used or not, the choice of utensils, patient positioning, and classification scales, which have already been described in the literature. If we remain entrenched in the same discussions, we will not only be stagnant but also overlook the potential for advancement in an area that still has much to offer regarding clinical reasoning, therapeutic tests, and individualized intervention planning. It is in this still unexplored space (i.e., questions that have not yet been formulated about clinical reasoning and decision-making) that the true FEES advancement lies, rather than repeatedly reproducing already known answers about the performance of the exam.

Findings such as posterior oral spillage, pharyngeal residues, penetration, and aspiration should not only be described or classified using scales that quantify their severity. In this way, the discussion still remains an observation that values the bolus rather than the physiology of swallowing . Such findings need to be interpreted by a swallowing specialist and related to the underlying pathophysiological mechanisms to support more informed decision-making based on the patient's profile^(9)^. In conclusion, no examination in dysphagic patients is conclusive, as these assessments show what happened but do not explain why. Therefore, the discussion on FEES should move beyond aspects related to the bolus, allowing for a better understanding of the underlying causes of the findings and providing stronger support for clinical decision-making in dysphagia.
